# Lipid moieties of *Mycoplasma pneumoniae* lipoproteins are the causative factor of vaccine-enhanced disease

**DOI:** 10.1038/s41541-020-0181-x

**Published:** 2020-04-08

**Authors:** Arlind B. Mara, Tyler D. Gavitt, Edan R. Tulman, Steven J. Geary, Steven M. Szczepanek

**Affiliations:** Department of Pathobiology and Veterinary Science and the Center of Excellence for Vaccine Research, University of Connecticut, 61 North Eagleville Road, Unit 3089, Storrs, CT 06269 USA

**Keywords:** Bacteriology, Bacterial host response, Infection, Infectious diseases, Vaccines

## Abstract

Vaccine-enhanced disease (VED) occurs as a result of vaccination followed by infection with virulent *Mycoplasma pneumoniae*. To date VED has prevented development of an efficacious vaccine against this significant human respiratory pathogen. Herein we report that vaccination of BALB/c mice with *M. pneumoniae* lipid-associated membrane proteins (LAMPs) induces lung lesions consistent with exacerbated disease following challenge, without reducing bacterial loads. Removal of lipid moieties from LAMPs prior to vaccination eliminates VED and reduces bacterial loads after infection. Collectively, these data indicate that lipid moieties of lipoproteins are the causative factors of *M. pneumoniae* VED.

## Introduction

*Mycoplasma pneumoniae (Mp)* is a highly contagious and widespread human respiratory pathogen causing over 2 million cases of community-acquired pneumonia (CAP) and ~100,000 adult hospitalizations annually in the United States^1^. Additionally, innate resistance to commonly used β-lactam antibiotics, increasing resistance to macrolide antibiotics, and mortality in certain demographic groups establish *Mp* as a high-risk pathogen capable of causing acutely severe disease^2–7^. Critically, no vaccine against *Mp* is available, as experimental vaccine-enhanced disease (VED) after infection has stymied further development.

*M. pneumoniae* VED was first reported in human volunteers in the 1960s. In these studies, prisoners and military recruits were vaccinated with formalin-inactivated or a serially passaged live-attenuated strain of *Mp*, then subsequently challenged with a virulent strain of the bacterium. While some protection was observed, many vaccinated individuals in both studies exhibited more severe clinical symptoms than those receiving a placebo control^8,9^. Recent studies by our group (and others) have since recapitulated VED in an animal model by utilizing live-attenuated or crude extract *Mp* vaccine candidates, respectively^10–13^. In these murine models, VED is characterized by more severe histopathology post-challenge, when compared to sham vaccinated, *Mp* challenged mice. VED has also been observed in vaccine candidates against other *Mycoplasma* species, making the identification of the causative factors a crucially important task for vaccine development against these atypical pathogens.

## Results and discussion

### Lipoproteins are the most abundant components of the lipid- associated membrane protein (LAMP) fraction

**Fig. 1:**
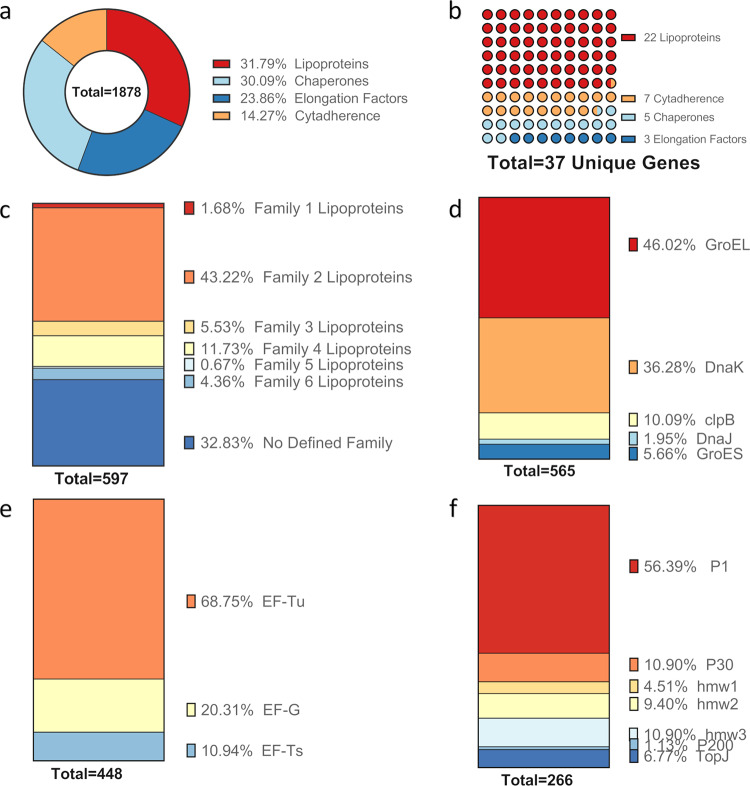
Lipoproteins are the most abundant immunogenic and antigenic components of the *Mp* LAMP fraction. **a** Breakdown of the major immunogenic/antigenic components of the LAMPs fraction as percentage of spectral hits. **b** Breakdown of the major immunogenic/antigenic components of the LAMPs fraction into number of unique genes represented in the fraction. Percent breakdown of **c** lipoproteins, **d** chaperones and chaperonins, **e** elongation factors, and **f** cytadherence-associated proteins represented in the LAMPs fraction.

To identify the specific causative factor(s) for *Mp* VED, we utilized TX-114 phase partitioning to fractionate *Mp* proteins into three fractions: a *LAMP* hydrophobic detergent phase, an aqueous phase (*Aq*) harboring mostly hydrophilic cytosolic proteins, and an insoluble phase (*Ins*) containing the insoluble triton shell and a spontaneously forming, phospholipid-rich precipitate^14^. An initial pilot study indicated that vaccination of BALB/c mice with *LAMPs* resulted in VED after challenge with virulent *Mp*. We performed proteomic analysis to reveal lipoproteins, elongation factors, chaperones and chaperonins, and cytadherence proteins (previously found to induce strong antibody responses^15–17^) as the most abundant immunogenic and antigenic components of the LAMP fraction (Fig. 1a–f). Of these, lipoproteins are the most abundant (31.79%) (Fig. 1a) and include twenty-two lipoproteins representing all six *Mp* lipoprotein families (Fig. 1b, c). Family 2 lipoproteins, paralogs of the immunodominant *Mycoplasma gallisepticum* nucleotide-binding virulence factor MslA^18,19^, were the most abundant LAMP lipoproteins, making up 43.22% of the lipoprotein fraction (Fig. 1c). Lipoproteins not belonging to a multigene family were the second most abundant lipoproteins in the lipoprotein fraction (32.83%), followed by Family 4 lipoproteins (11.73%), Family 3 lipoproteins (5.53%) then Family 6 lipoproteins (4.36%) (Fig. 1c). Members of Family 5 lipoproteins (which are specific to *Mp*) were also identified in the fraction, albeit at minute relative quantities (0.67%) (Fig. 1c). Mycoplasma lipoprotein lipid moieties are known to be potent immuno-stimulators, inducing expression of inflammatory cytokines such as TNF-α, IL-6, and IL-1β following the recognition of their lipid moieties by Toll-like receptor complexes^20^. Given that these cytokines are frequently associated with immunopathology, we hypothesized that sensitization by *Mp* lipoprotein lipid moieties during vaccination induces VED upon challenge with virulent *Mp*.

### Lipoprotein lipid moieties are responsible for VED

**Fig. 2:**
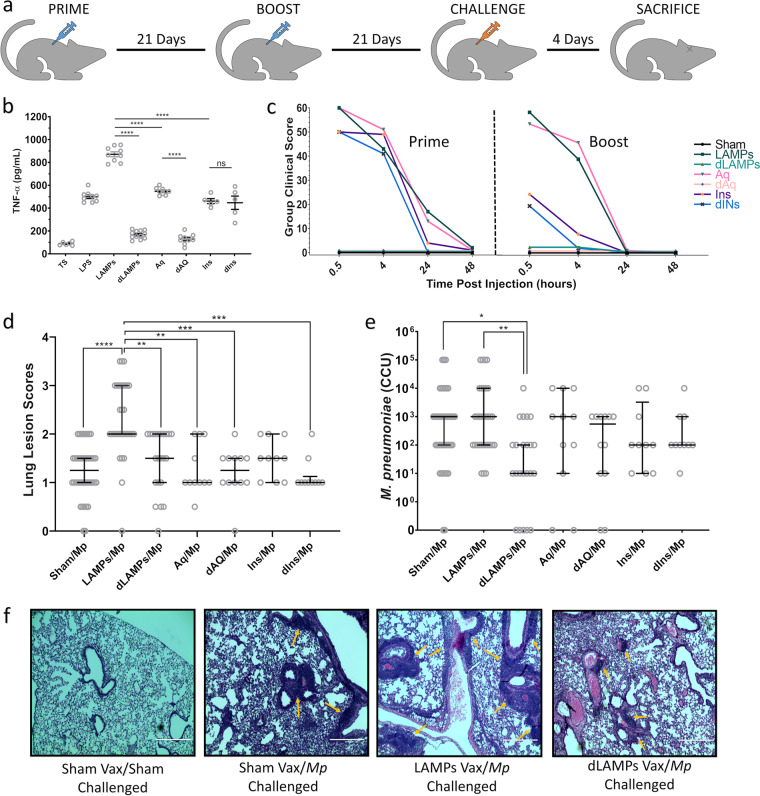
Vaccination with native *M. pneumoniae* lipoproteins, but not delipidated lipoproteins induces vaccine-enhanced disease after challenge. **a** Illustration of experimental timeline. **b** Supernatant TNF-α levels produced by murine J774A.1 macrophages stimulated by intact or lipase treated *M. pneumoniae* fractions. Each individual point represents an individual biological replicate. **c** Group clinical scores of mice showing clinical signs such as piloerection, hunching, nose bulge, orbital tightening and lethargy as a function of time after prime and boost injections. Group clinical scores were derived by adding +1 per sign displayed per animal. Data from one individual experiment with *n* = 12 animals per group. **d** Histopathological lung lesion scores and **e** bacterial loads recovered from vaccinated-then-challenged animals. Data for (**d**), and (**e**) are pooled from five independent experiments, with each point representing data from an individual animal. **f** Representative histopathological lung images of sham vaccinated/sham challenged animals (0), sham vaccinated*/M. pneumoniae* challenged animals (1.5), *LAMPs* vaccinated/*M. pneumoniae* challenged animals (3), and d*LAMPs* vaccinated/*M. pneumoniae* challenged animals (1.5). Arrows point to perivascular and peribronchiolar leukocytic infiltrates. Error bars indicate mean and standard error of the mean (SEM) for parametric data (**b**) and median and interquartile ranges (IQR) for non-parametric data (**d**, **e**) (*p < 0.05, **p < 0.01, ***p < 0.001, ****p < 0.0001).

To test our hypothesis, TX-114-derived *Mp* fractions were treated with exogenous lipoprotein lipase to generate delipidated fractions (*dLAMPs*, *dAq*, and *dIns*), which could be tested for immuno-stimulation in vitro and as vaccine candidates in vivo. Successful delipidation was assessed via a TLR2 bioassay. In murine macrophages, *LAMPs* stimulated higher levels of TNF-α than *Aq* and *Ins* fractions. Delipidation significantly reduced LAMP-stimulated TNF-α production, notably to levels lower than those stimulated by *Aq* or *Ins* fractions (Fig. 2b). Surprisingly, delipidation also reduced *Aq* fraction-stimulated TNF-α production, suggesting that some lipoproteins may have sequestered to the *Aq* phase during TX-114 phase partitioning. Delipidation did not affect the ability of the *Ins* fraction to stimulate TNF-α production in vitro (Fig. 2b).

To test the effect of lipoprotein delipidation on VED, native and delipidated *Mp* fractions (*LAMPs, dLAMPs, Aq, dAq, Ins*, and *dIns*) were used to intraperitoneally vaccinate mice according to the schedule illustrated in Fig. 2a with 50 μg of protein from each fraction per dose. Shortly after vaccination, mice receiving non-lipase-treated fractions began to display clinical signs consistent with mild septic shock (Supplementary Table 1, Supplementary Fig. 1), which we quantified as a group clinical score (Fig. 2c). While these clinical signs abated by 48-h post-vaccination, they point to potential immediate safety concerns for vaccine candidates containing intact lipoproteins. Notably, early clinical signs were absent in mice vaccinated with delipidated fractions. Given that in vivo data mirrored those obtained in vitro (Fig. 2b), early clinical signs may have resulted from the over-stimulation of peritoneal immune cells by the lipid moieties of native *Mp* lipoproteins.

In terms of VED, only mice vaccinated with *LAMPs* (containing native lipoproteins) exhibited histopathological lung lesions that were more severe than sham-vaccinated/challenged animals, consistent with LAMP-mediated VED (Fig. 2d, f). Furthermore, vaccination with *LAMPs* did not reduce bacterial loads, suggesting that the induced inflammation is solely pathologic and consistent with VED (Fig. 2e). Importantly, vaccination with delipidated LAMPs (*dLAMPs)* did not exacerbate lung pathology (Fig. 2d, f), demonstrating that the factors responsible for VED are the lipid moieties of *Mp* lipoproteins. Compared to controls, *dLAMP*-vaccinated mice also had significantly lower bacterial loads (Fig. 2e). These data suggest that the *dLAMP* vaccine preparation includes protective antigens that improve bacterial clearance and can reduce *Mp*-induced lung disease when freed of the VED effects associated with *Mp* lipoprotein lipid moieties.

Mice vaccinated with MPLA-SM adjuvanted *dLAMPs* and challenged had significantly reduced lung lesion scores when compared to sham vaccinated, *Mp* challenged mice (Supplementary Fig. 2A, C), but did not differ in bacterial loads recovered (Supplementary Fig. 2B). This was unsurprising as lesion scores did not always correlate with bacterial loads. These data further indicate that the choice of adjuvant will be critical in developing a vaccine that reduces bacterial loads while also protecting the host from unnecessary pulmonary inflammation.

## Conclusions

The identification of *Mp* lipoprotein lipid moieties as the cause of *Mp* VED is a breakthrough for the field, overcoming a major roadblock for *Mp* vaccine development. The findings presented here may be broadly applicable to other *Mycoplasma* species for which vaccine induced disease exacerbation has been reported. Vaccination of calves with inactivated whole cell vaccines and partially purified membrane proteins of *Mycoplasma bovis* resulted in increased pulmonary pathology upon virulent challenge^21,22^. Additionally, vaccination of cattle with inactivated whole cell *Mycoplasma mycoides* subsp. *mycoides SC* and a subunit lipoprotein Q (LppQ) vaccine also were reported to exacerbate the effects of Contagious Bovine Pleuropneumonia (CBPP) in challenged animals^23^. These studies did not identify the lipid moieties of lipoproteins or any other factors as the causative factors of VED in these cases. Given the parallels of these studies with our data, however, it is attractive to speculate that the lipid moieties of the lipoproteins included in these vaccine candidates may have been the maladaptive factors responsible for disease exacerbation. Indeed, vaccination of cattle with the N terminus of LppQ (which includes the region where the lipid moieties attach) failed to be protective and was also associated with adverse events^24^. It is important, however, to note that more studies need to be conducted to demonstrate the maladaptive effect of lipoproteins in ruminant mycoplasmosis vaccines.

In terms of *M. pneumoniae*, our data indicate that caution should be used when considering the inclusion of native Mycoplasma lipoproteins in future vaccine formulations against this significant human pathogen. Furthermore, the findings here may be broadly applicable to other bacterial pathogens for which VED is observed, and suggest that the potential role of lipoproteins in this phenomenon should not be easily dismissed.

## Methods

### TX-114 phase partitioning of *Mp* proteins

TX-114 partitioning^25^ of *M. pneumoniae* proteins was performed as detailed in the supplementary methods (see Extended Methods). Fractions were ethanol precipitated and resuspended in TS buffer (15 mM Tris, 150 mM Nacl, pH 7.6)via sonication (Sonics VCX-500 ultrasonicator, Newtown, CT), then quantified by a Qubit 2.0 Fluorometer (Thermofisher, Waltham, MA) protein assay kit.

### Proteomic analysis of *Mp LAMPs*

Purified LAMPs were prepared using a slightly modified filter-aided sample preparation (FASP) method^26^ described in the supplementary methods (see Extended Methods). Purified peptides were injected onto a PepMap RSLC C18 column (Thermo Scientific) and separated using reversed phase gradient on a Dionex Ultimate 3000 RSLC UPLC instrument (Thermo Scientific) coupled directly to a Q Exactive HF mass spectrometer (Thermo Scientific) via electrospray ionization. MaxQuant (v1.6.1.0) was used to search raw files against a custom *Mp* proteome database (CP010538_faa) using the Andromeda search engine^27^ and for peptide and protein quantification using the LFQ algorithm. Scaffold v4.9 (Proteome Software, Inc.) was used for visualization and analysis, and gene designations were identified through comparison to the *Mycoplasma pneumoniae* (T00006) KEGG genome database with 95–100% sequence identity and lowest E-value used as cut-offs. Multigene *Mp* lipoproteins were categorized into the 6 numerically named families based on previously established criteria^28^. Lipoproteins not belonging to a multigene family were categorized as “No Defined Family”.

### Lipoprotein lipase digestion and macrophage inflammatory bioassay

Precipitated *Mp* TX-114 fractions (Insoluble: *Ins*, Aqueous: *Aq*, and Detergent: *LAMPs*) were treated with Lipoprotein Lipase from *Burkholderia sp*. (EC 3.1.1.34; Sigma Aldrich, St. Louis, MO) to generate the delipidated fractions (respectively). The efficiency of lipoprotein lipase treatment was assessed via TLR-2 Bioassay/Macrophage Inflammatory Assay^29^ described in the supplementary methods (see Extended Methods).

### Bacterial strains and animal studies

*Mycoplasma pneumoniae* strain PI1428 was utilized for all aspects of this study. All animal experiments were conducted in accordance with approved Institutional Animal Care and Use Committee protocol (A17–034) as described in the supplementary methods (see Extended Methods).

### Statistical analyses

Results were analyzed via a non-parametric one-way ANOVA on ranks (Kruskal–Wallis) with a Dunn’s post hoc test or a parametric ordinary one-way ANOVA with a Tukey’s post hoc test for multiple pairwise comparisons between groups. Analysis of data with only two groups was conducted utilizing a one tailed Mann–Whitney *U* test for nonparametric data (*α* = 0.05). All data were analyzed using the GraphPad Prism software, version 8.02 (GraphPad Software, La Jolla, California, USA).

### Reporting summary

Further information on experimental design is available in the Nature Research Reporting Summary linked to this article.

## Supplementary information

Supplementary Information

Reporting Summary

## Data Availability

The mass spectrometry proteomics data have been deposited to the ProteomeXchange Consortium via the PRIDE^30^ partner repository with the dataset identifier PXD016814. All other data are included in this manuscript and supplementary file.
