# Supplementary material for: Lipid moieties of Mycoplasma pneumoniae lipoproteins are the causative factor of vaccine-enhanced disease
Source: NPJ Vaccines. 2020 Apr 8;5:31. doi: 10.1038/s41541-020-0181-x (PMC7142147; doi:10.1038/s41541-020-0181-x)
Supplement: Supplementary file 1 — Supplementary Information [file 41541_2020_181_MOESM1_ESM.pdf]

SUPPLEMENTARY INFORMATION FOR:

Lipid moieties of *Mycoplasma pneumoniae* lipoproteins are the causative factor of vaccine-enhanced disease

Arlind B. Mara[1], Tyler D. Gavitt[1], Edan R. Tulman[1], Steven J. Geary[1,*], and Steven M. Szczepanek[1,*]

[1]Department of Pathobiology and Veterinary Science and the Center of Excellence for Vaccine Research, University of Connecticut, 61 N Eagleville Road, Unit 3089, Storrs, Connecticut 06269, USA.

*Corresponding authors. S.M.S: steven.szczepanek@uconn.edu
S.J.G: steven.geary@uconn.edu

Table of Contents

1) Supplementary Table 1…………………………………………………………………...3

2) Supplementary Figure 1………………………………………………………………..4

3) Supplementary Figure 2……………………………………………………….…..…..5

4) Extended Methods…………………………………………………...…………….6-13

PILOERECTION

	Hours Post Prime Injection				Hours Post Boost Injection			
**Group**	**0.5**	**4**	**24**	**48**	**0.5**	**4**	**24**	**48**
**Sham**	0/22	0/22	0/22	0/22	0/22	0/22	0/22	0/22
**LAMPs**	12/12	12/12	8/12	2/12	12/12	12/12	0/12	0/12
**dLAMPs**	0/12	0/12	0/12	0/12	1/12	1/12	0/12	0/12
**Aq**	12/12	12/12	11/12	1/12	11/11*	11/11*	1/11*	0/11*
**dAq**	0/12	0/12	0/12	0/12	0/12	0/12	0/12	0/12
**Ins**	10/10	10/10	4/10	1/10	10/10	4/10	0/10	0/10
**dIns**	10/10	10/10	0/10	0/10	10/10	2/10	0/10	0/10

HUNCHING

	Hours Post Prime Injection				Hours Post Boost Injection			
**Group**	**0.5**	**4**	**24**	**48**	**0.5**	**4**	**24**	**48**
**Sham**	0/22	0/22	0/22	0/22	0/22	0/22	0/22	0/22
**LAMPs**	12/12	12/12	2/12	0/12	12/12	7/12	0/12	0/12
**dLAMPs**	0/12	0/12	0/12	0/12	0/12	0/12	0/12	0/12
**Aq**	12/12	9/12	0/12	0/12	11/11*	9/11*	0/11*	0/11*
**dAq**	0/12	0/12	0/12	0/12	0/12	0/12	0/12	0/12
**Ins**	10/10	10/10	0/10	0/10	5/10	0/10	0/10	0/10
**dIns**	10/10	7/10	0/10	0/10	2/10	0/10	0/10	0/10

ORBITAL TIGHTENING

	Hours Post Prime Injection				Hours Post Boost Injection			
**Group**	**0.5**	**4**	**24**	**48**	**0.5**	**4**	**24**	**48**
**Sham**	0/22	0/22	0/22	0/22	0/22	0/22	0/22	0/22
**LAMPs**	12/12	12/12	4/12	0/12	12/12	11/12	0/12	0/12
**dLAMPs**	0/12	0/12	0/12	0/12	1/12	1/12	0/12	0/12
**Aq**	12/12	12/12	2/12	0/12	11/11*	11/11*	0/11*	0/11*
**dAq**	0/12	0/12	0/12	0/12	0/12	0/12	0/12	0/12
**Ins**	10/10	10/10	0/10	0/10	10/10	4/10	0/10	0/10
**dIns**	10/10	10/10	0/10	0/10	8/10	0/10	0/10	0/10

NOSE BULGE

	Hours Post Prime Injection				Hours Post Boost Injection			
**Group**	**0.5**	**4**	**24**	**48**	**0.5**	**4**	**24**	**48**
**Sham**	0/22	0/22	0/22	0/22	0/22	0/22	0/22	0/22
**LAMPs**	12/12	12/12	3/12	0/12	12/12	7/12	0/12	0/12
**dLAMPs**	0/12	0/12	0/12	0/12	0/12	0/12	0/12	0/12
**Aq**	12/12	9/12	5/12	0/12	11/11*	11/11*	0/11*	0/11*
**dAq**	0/12	0/12	0/12	0/12	0/12	0/12	0/12	0/12
**Ins**	10/10	10/10	0/10	0/10	0/10	0/10	0/10	0/10
**dIns**	10/10	7/10	0/10	0/10	0/10	0/10	0/10	0/10

LETHARGY

	Hours Post Prime Injection				Hours Post Boost Injection			
**Group**	**0.5**	**4**	**24**	**48**	**0.5**	**4**	**24**	**48**
**Sham**	0/22	0/22	0/22	0/22	0/22	0/22	0/22	0/22
**LAMPs**	12/12	6/12	0/12	0/12	12/12	3/12	0/12	0/12
**dLAMPs**	0/12	0/12	0/12	0/12	0/12	0/12	0/12	0/12
**Aq**	12/12	9/12	0/12	0/12	11/11*	5/11*	0/11*	0/11*
**dAq**	0/12	0/12	0/12	0/12	0/12	0/12	0/12	0/12
**Ins**	10/10	9/10	0/10	0/10	0/10	0/10	0/10	0/10
**dIns**	10/10	7/10	0/10	0/10	0/10	0/10	0/10	0/10

Supplementary Table 1. Numbers of mice per group displaying clinical signs at 0.5, 4, 24, and 48 hours post prime and boost injections. *One mouse from this group was found dead 8 days after primary vaccination for unrelated reasons and was excluded from further analysis.

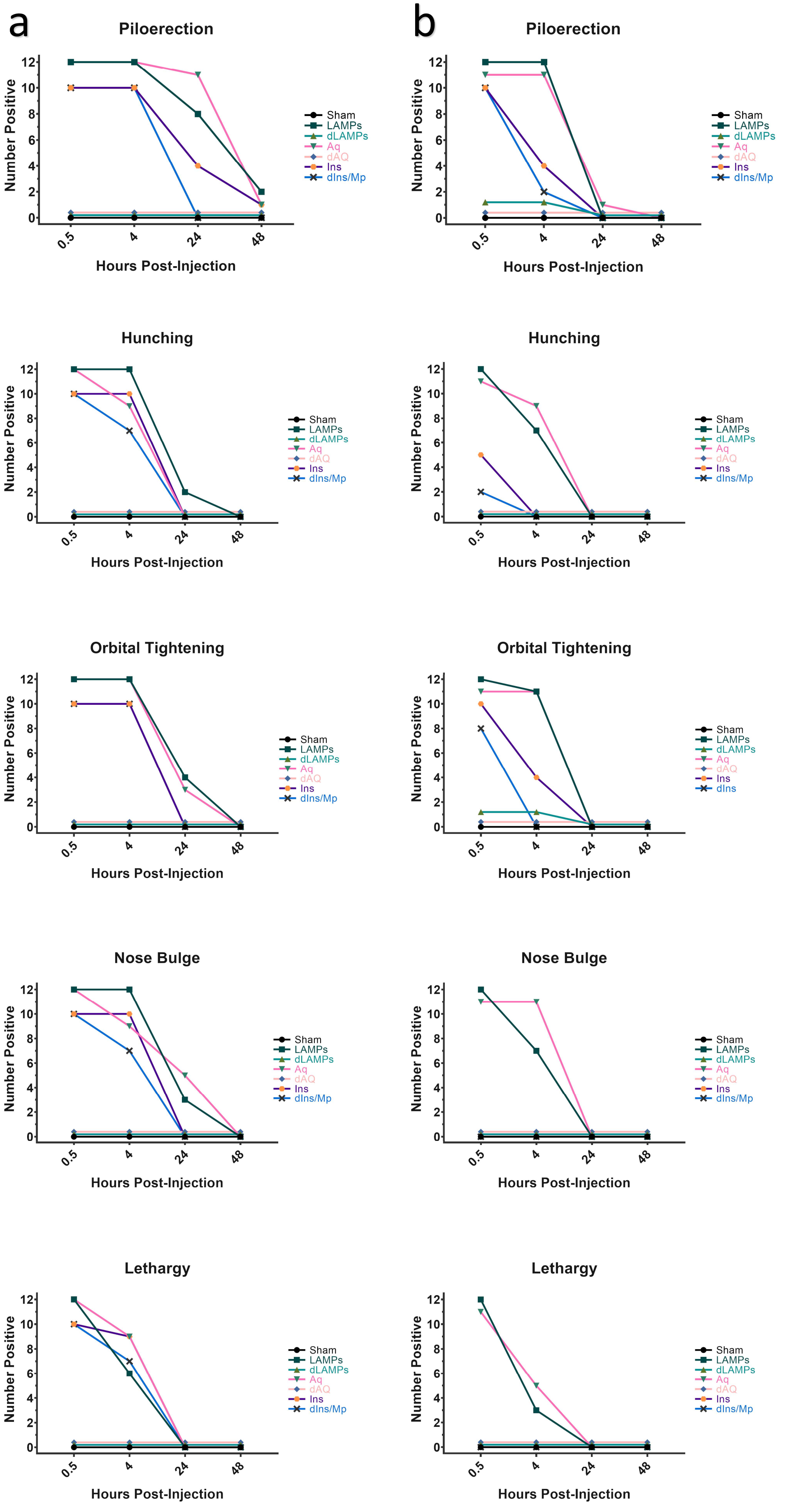

Supplementary Figure 1. Numbers of vaccinated animals showing clinical signs after Prime **(A)** injection and Boost **(B).**

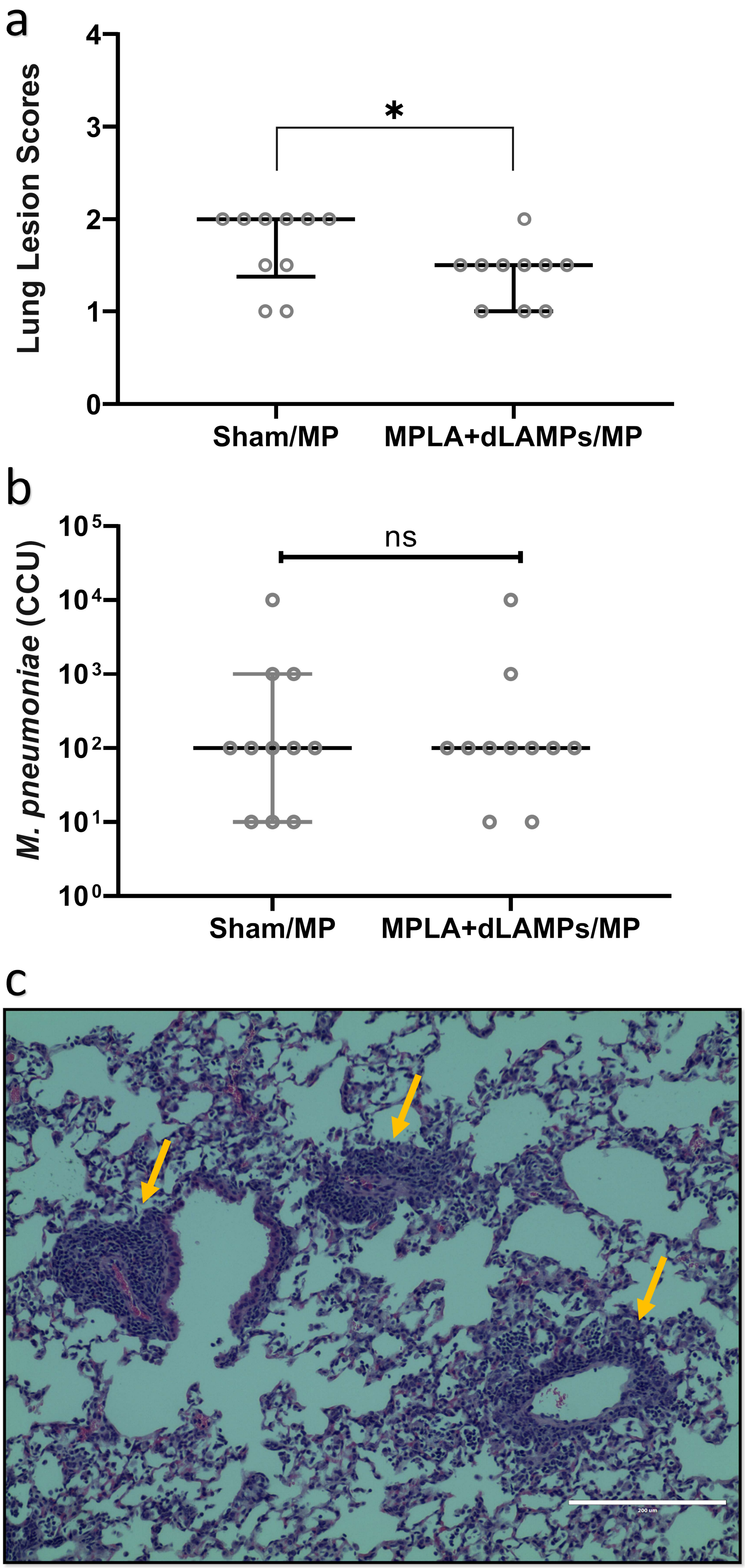

Supplementary Figure 2. Histopathological lung lesion scores **(A)** and bacterial loads **(B)** recovered from vaccinated then challenged animals. **(C)** Representative lung histopathology image from an MPLA+dLAMPs vaccinated, *Mp* challenged mouse receiving a lesion score of 1.5 (median score of the group). Arrows indicate perivascular and peribronchiolar infiltrates. Photo taken at **100x** magnification on a digital Evos XL Microscope (Life Technologies). White bar = 400µm. *Error bars indicate median and interquartile ranges (IQR) (* $p<0.05$, ** $p<0.01$, *** $p<0.001$, **** $p<0.0001$).*

Extended Methods

TX-114 Phase Partitioning of *M. pneumoniae* proteins

Mycoplasma pneumoniae LAMPs were extracted utilizing TX-114 partitioning using a slightly modified protocol from the one established by Bordier (1981). *M. pneumoniae* PI1428 cells were cultured in T-175 cell culture flasks at 37ºC in complete FC medium until mid-log phase as determined by acid-mediated shift of phenol red dye from red to orange. Adherent bacterial cells were scraped onto the medium then pelleted by centrifugation. The pellet was washed with PBS then solubilized in 5mL of TS-EDTA buffer (20mM Tris, 150mM NaCl, 5mM EDTA pH 7.6) containing 1mM PMSF (protease inhibitor) and 2% (v/v) TX-114. The solution was rocked for 2 hours at 4ºC, followed by centrifugation at 10,000xg at 4ºC for 10 minutes to pellet the insoluble phase. The soluble phase was transferred to a new tube and incubated at 37ºC for until solution became cloudy (indicating condensation of detergent micelles), then centrifuged for 15 minutes at room temperature to separate the detergent and aqueous phases which were then aliquoted into new tubes. Appropriate amounts of TS-EDTA buffer and TX-114 detergent were added to the tubes containing the insoluble, aqueous and detergent fractions to reach a 2% TX-114 solution, and the phase partitioning was repeated twice to clean the fractions.

LAMP sample preparation using FASP methods

Purified LAMPs were prepared using a slightly modified filter-aided sample preparation (FASP) method in a Microcon YM-10 10 kD molecular weight cutoff (MWCO) filter (Thermo Fisher Scientific) (29). Briefly, samples were suspended in UA buffer and reduced for 1.5 hr at 37°C using 25 mM dithiothreitol in UA buffer. Samples were spun at 14,000 x g for 40 min, washed with 200 µL UA buffer and spun at 14,000 x g for 40 min. Samples were alkylated using 50 mM iodoacetamide in UA buffer for 15 min in the dark at 37°C. After a 14,000 x g for 30 min spin, the samples were washed twice with 100 µL UB buffer with identical centrifugation cycles, the MWCO filters were washed with 50 µL

UB buffer and protein was placed in a clean 1.5 mL Eppendorf tube. The filter was washed twice more with 50 µL aliquots of 0.1 M ammonium bicarbonate and pooled. Endoproteinase LysC (Pierce) was added at a 1:50 enzyme:protein ratio and left to digest at 37°C for 16 hr. Samples were diluted to <1 M urea with 0.1M ammonium bicarbonate, sequencing grade modified trypsin (Promega) was added at a 1:50 enzyme:protein ratio and left to digest for an additional 8 hr at 37°C. Proteolysis was quenched using formic acid and the resulting peptides were desalted using C18 Peptide Desalting Spin Columns (Pierce) per manufacturer's instructions.

Lipoprotein Lipase Digestion and Macrophage Inflammatory Bioassay

Precipitated *M. pneumoniae* TX-114 fractions (Insoluble: *Ins*, Aqueous: *Aq*, and Detergent: *LAMPs*) were treated with 2500 units of Lipoprotein Lipase from *Burkholderia sp*. (EC 3.1.1.34; Sigma Aldrich, St. Louis, MO) per 2µg of protein, for 48 hours at 37ºC with shaking at 250 RPM on an orbital shaker to generate the delipidated fractions called *dIns, dAq*, and *dLAMPs*, respectively. The efficiency of lipoprotein lipase treated was assessed via a Macrophage Inflammatory Bioassay () with some modifications. Briefly, murine J774A.1 macrophages were stimulated for 6 hours with 20ug of protein from each TX-114 fraction and its delipidated counterpart or 15ug of LPS as a positive control for macrophage activation. Supernatant TNF-α levels were measured using a commercial murine TNF-α sandwich ELISA (Biolegend, San Diego, CA) according to manufacturer's instructions.

Bacterial Strains and Inoculum Culture Conditions

Mycoplasma pneumoniae strain PI1428 was utilized for all aspects of this study. For infection studies, frozen 50µL aliquots of low passage (P12-P13) *M. pneumoniae* PI1428 were thawed and resuspended in in 10mL of complete Fortified Commercial (FC) medium (20% heat inactivated horse serum, 5% yeast extract). Cultures were incubated at 37ºC with orbital shaking at 120 RPM. After 5 hours, optical density at 620 nm (OD_{620}) was used to estimate colony forming units (CFU) counts per mL of culture. Furthermore, color changing unit (CCU) measurements were conducted using 10-fold

serial dilutions to validate spectrophotometric estimations. Samples were then centrifuged at 2000xg for 10 min at 4ºC, the supernatant decanted, and the pellet suspended to the desired concentration in fresh complete FC medium.

***In vivo* Vaccination/Challenge Studies**

All animal experiments were conducted in accordance with our approved Institutional Animal Care and Use Committee protocol (A17-034). Male and Female Specific Pathogen Free (SPF) BALB/c mice (8 weeks old) were purchased from Jackson Laboratories (Bar Harbor, ME) and allowed to acclimate for 1 week prior to use. For vaccination, mice were anesthetized using vaporized isoflurane and intraperitoneally injected with 250µL of sterile physiological saline (0.9%) for sham vaccination, or 250µL containing 50µg of protein from the appropriate fraction. For mice receiving adjuvanted MPLA-SM + dLAMPs treatement, mice were injected with 250µL of sterile physiological saline containing 20µg MPLA-SM + 50 µg of dLAMPs. Mice were boosted similarly 21 days after the primary injection. Mice showing clinical signs after injections were closely observed for 48 hours and clinical signs such as piloerection, hunching, nose bulge, orbital tightening, and lethargy were recorded by counting the number of animals per group displaying said clinical signs. Group clinical scores were derived by adding +1 per sign displayed, per animal. Max clinical score per group can be calculated by (n*5) where n is the number of animals per group and 5 is the number of categories of clinical signs recorded. Twenty-one days after the boost dosage, mice were anesthetized using vaporized isoflurane and intranasally inoculated with 50µL of FC medium containing $1x10^{8}$ CFU *M. pneumoniae* PI1428. Four days post-infection, mice were humanely sacrificed via cervical dislocation and lungs were immediately harvested for histopathology and *Mycoplasma* recovery.

Mycoplasma Recovery and Histopathology

For *Mycoplasma* recovery, the lower right lobe of the lung was removed and placed into 3mL of FC medium, vortexed, and incubated for 3 hours at 37ºC. The remaining lung tissue was inflated with 10% neutral buffered formalin and allowed to fix for histopathologic evaluation. After 3 hours of incubation, *Mycoplasma* recovery samples were passed through a 0.45μm filter and transferred to new sterile tubes containing FC medium. Quantification of recovery cultures was performed by assessing Color Changing Units (CCUs) in 10-fold serial dilutions performed on 96 well tissue culture plates. Samples were incubated for 28 days, and color change was observed and recorded daily. After fixation for 48 hours in 10% neutral buffered formalin, tissues were routinely processed and stained. Perivascular and peribronchiolar leukocytic infiltrates were graded in increasing severity with scores 0 (no visible lesions), 1 (mild lesions), 2 (moderate lesions), 3 (marked lesions), and 4 (severe lesions), with half-step intervals (i.e. +0.5) indicating lesions that fall between two categories. Lesion score evaluations were performed in a blinded fashion by an experienced individual trained in the evaluation of these specific pathologic lesions by a board-certified pathologist. Scores were assigned based on the density of the perivascular and peribronchiolar infiltrates and the following objective criteria:

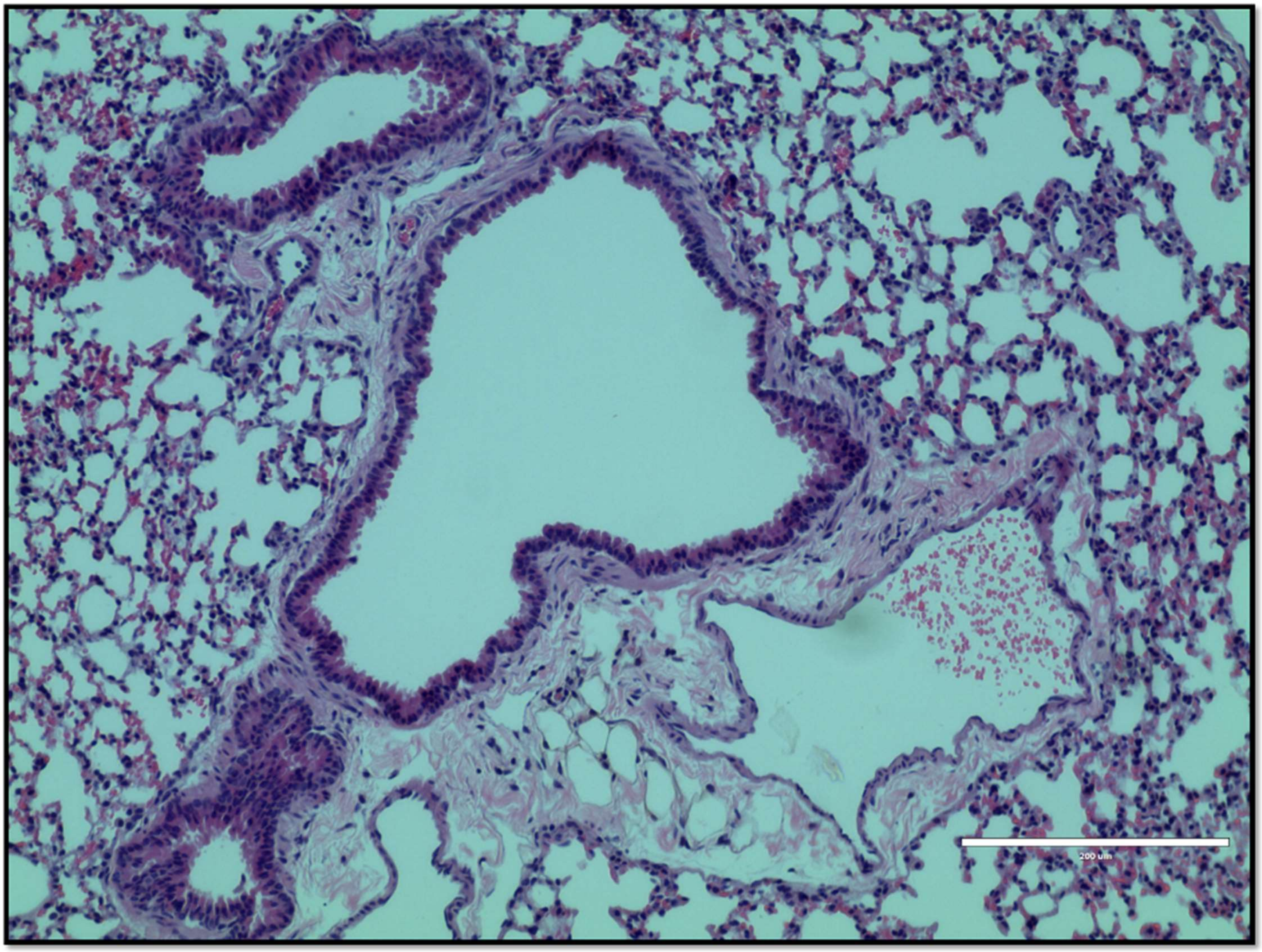

Lesion Score 0: No visible peribronchiolar and perivascular leukocytic infiltrates observed. Representative histological image of an H&E stained murine lung cross-section with a lesion score of 0. Photo taken at **200x** magnification on a digital Evos XL Microscope (Life Technologies).
White bar = 200μm.

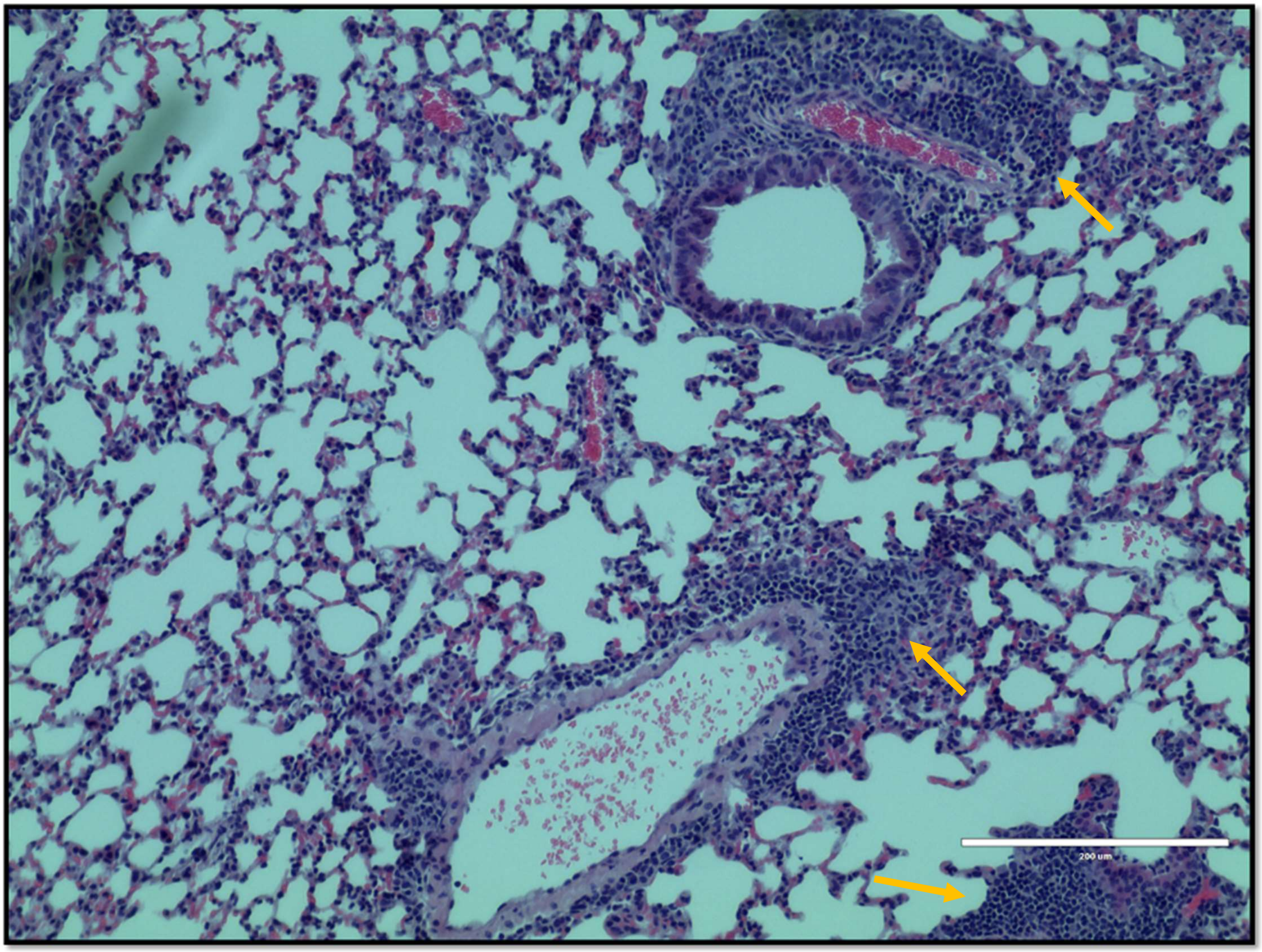

Lesion Score 1: Perivascular and peribronchiolar leukocytic infiltration with no consistent cuffing of airways or vessels by the esion and no spillover of a lesion to nearby airways or vessels. Representative histological image of an H&E stained murine lung cross-section with a lesion score of 1. Arrows indicate perivascular and peribronchiolar infiltrates. Photo taken at **200x** magnification on a digital Evos XL Microscope (Life Technologies). White bar = 200µm.

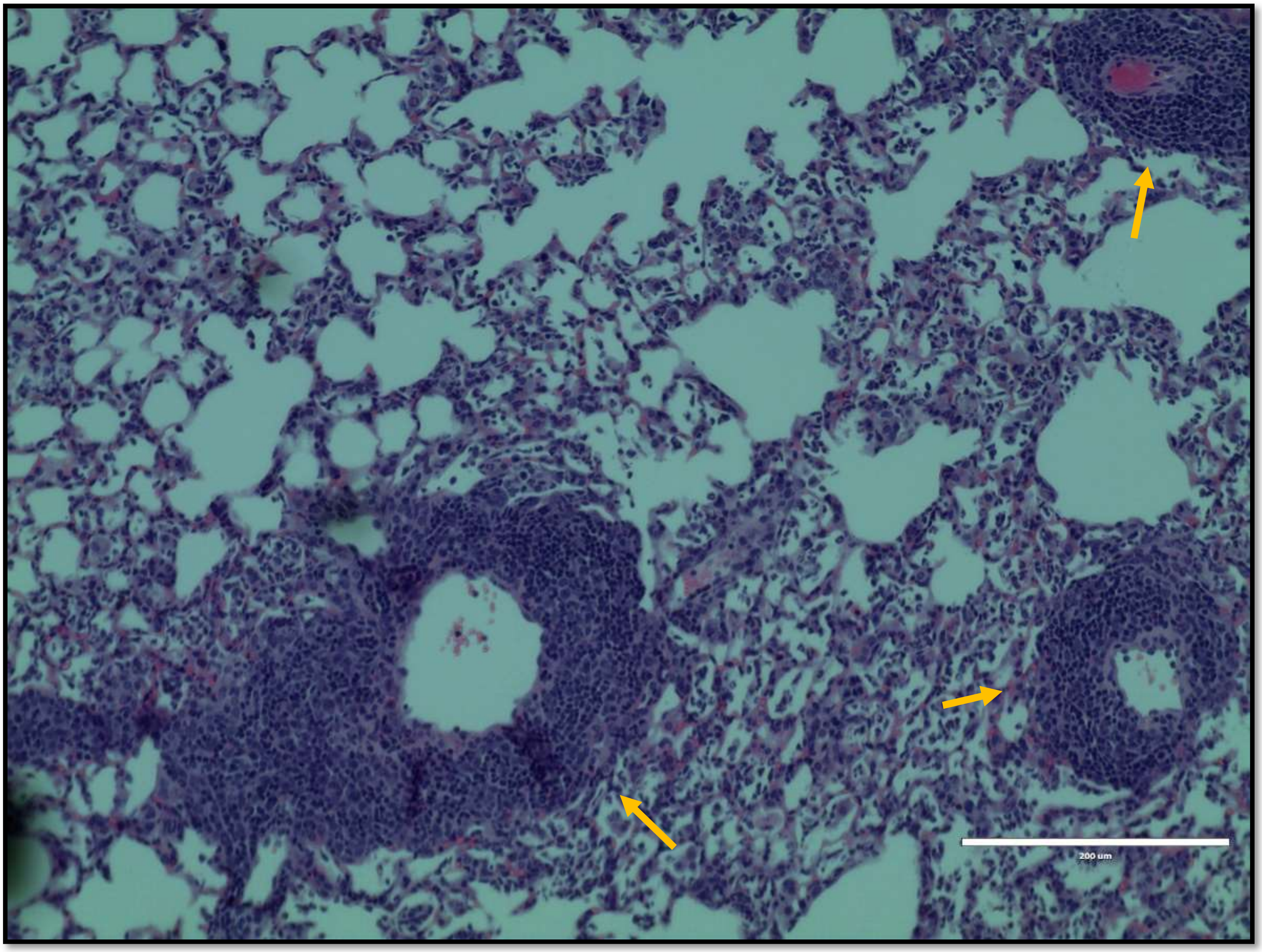

Lesion Score 2: Perivascular and peribronchiolar leukocytic infiltration with cuffing of airways or vessels **but no** marked spillover of lesions to nearby airways and vessels.
Representative histological image of an H&E stained murine lung cross-section with a lesion score of 2. Arrows indicate perivascular and peribronchiolar infiltrates. Photo taken at **200x** magnification on a digital Evos XL Microscope (Life Technologies). White bar = 200μm.

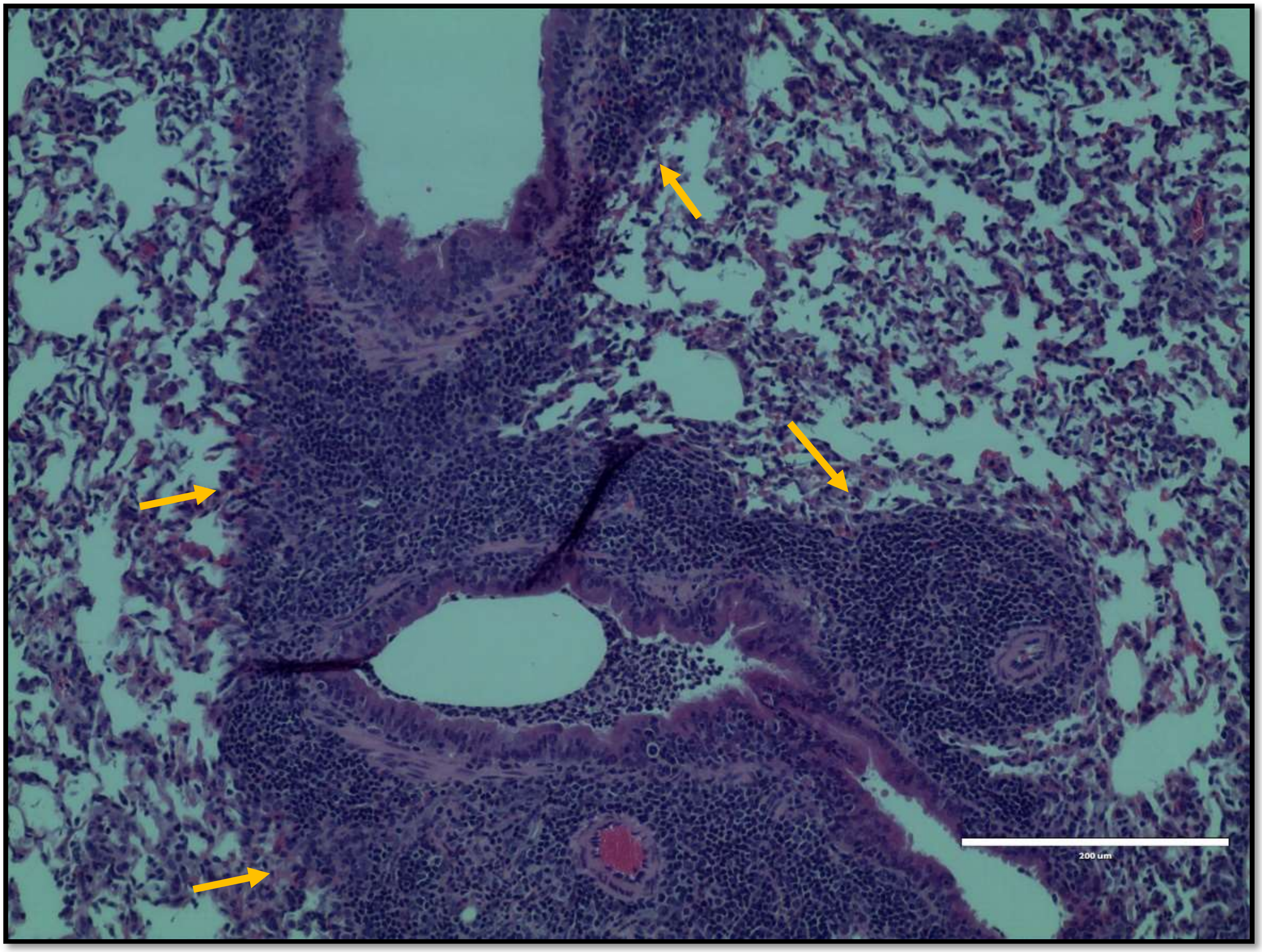

<u>Lesion Score 3:</u> Perivascular and peribronchiolar leukocytic infiltration with cuffing of airways or vessels **and** at least one instance of lesion spillover to nearby airways or vessels. Representative histological image of an H&E stained murine lung cross-section with a lesion score of 3. Arrows indicate perivascular and peribronchiolar infiltrates. Photo taken at **200x** magnification on a digital Evos XL Microscope (Life Technologies). White bar = 200μm.

<u>Lesion Score 4:</u> Consistent perivascular and peribronchiolar leukocytic infiltration with cuffing of airways and blood vessels and consistent lesion spillover to nearby airways or vessels. No representative image could be provided for a lesion score of 4 as no animals in the experiments performed in this study met the criteria established for this score.
